# Graphene Oxide‐Based Sensor for Ultrasensitive Visual Detection of Fluoride

**DOI:** 10.1002/advs.201600217

**Published:** 2016-07-21

**Authors:** Tapas K. Mandal, Yi Hou, Zhenyu Gao, Haoran Ning, Wensheng Yang, Mingyuan Gao

**Affiliations:** ^1^ Institute of ChemistryChinese Academy of SciencesBei Yi Jie 2Zhong Guan CunBeijing100190China; ^2^ School of Chemistry and Chemical EngineeringUniversity of Chinese Academy of SciencesBeijing100049China; ^3^ College of ChemistryJilin UniversityChangchun100032China

**Keywords:** fluorescence quenching, fluorescence reactivation, nitrogen‐doped fluorescent graphene oxide, ultrasensitive F^−^ ion detection, XPS analysis

## Abstract

**Visual fluoride ion detection** with a detection limit down to pmol L^−1^ is achieved through quenching/reactivating the fluorescence of N‐doped graphene oxide.

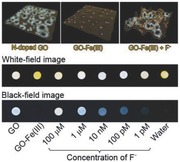

Fluoride plays an important role in human health. An appropriate amount of fluoride is essential to dental care and clinical treatment for osteoporosis.^1^ However, excess fluoride is not only toxic to aquatic organisms and plants,^2^ but also causes serious health problems to human beings, such as acute gastric and kidney disorders, dental and skeletal fluorosis,^3^ and DNA damage.^4^ In fact, ground waters with high fluoride concentrations occur in many areas of the globe including large parts of Africa, China, Southern Asia (India, Sri Lanka), and the Eastern Mediterranean, putting hundreds of millions of population at risk.^5^ Therefore, developing facile and sensitive fluoride detection methods suitable for monitoring F^−^ and its movement in drinking water system is of great importance and research value. Nevertheless, fluoride occurs as the most electronegative element. Its high hydration enthalpy and very comparable size with hydroxide ion set great challenges to achieve sensitive fluoride sensing as well as quantitative determination over a large concentration range.

Until now, ion‐chromatography, ion‐selective electrode, and different colorimetry methods are most commonly used for quantitative analysis of fluoride.^6^ Upon ion‐chromatography, fluoride separated from the other anions is determined by the conductivity of the eluent. But water eluted first normally produces a negative peak which partly cancels the positive signal of low concentration fluoride anion, and simple organic acids eluting close to fluoride also cause interference. Therefore, the sensitivity of ion‐chromatography in detecting low concentration fluoride remains a problem to solve. The ion‐selective electrode method relies on the conversion of the activity of F^−^ into an electrical potential through a fluoride‐selective electrode. Since the fluoride ion activity depends on the total solution ionic strength and pH, an appropriate buffer is essentially required to exclude the interference of hydroxyl ion, offer a nearly uniform ionic strength background, and break up fluoride complexes formed with polyvalent cations such as Si^4+^, Fe^3+^, and Al^3+^. In this context, the ion‐selective electrode method is facing reproducibility problem. The colorimetry methods determine F^−^ ion through colorimetric reactions between F^−^ and metal–organic complexes at controlled pH, but the sensitivity and reproducibility are not satisfying either.

The significance of fluoride therefore has motivated the development of new fluoride sensing methods over the past decades. Among various techniques, fluorescence sensors are the most attractive due to their high sensitivity and low detection limits.^7^ Until now, a large number of fluoride fluorescence sensors reported are based on the deprotonation of C—H bond^8^ or pyrozole N—H^9^ of different types of organic dyes, or fluoride‐induced desilylation reactions.^10^ Detection limits down to μmol L^−1^ have been achieved. Bein and co‐workers recently reported a different type of turn‐on sensors by entrapping fluorescein as reporter within a metal–organic framework (MOF).^11^ Upon the metal–anion complexation induced decomposition of the MOF framework, fluoride detection is achieved. Although HCO_3_^−^ exhibits a remarkable interference apart from SO_4_^2−^ and AcO^−^, detection limit close to 1 μmol L^−1^ is achieved. Kumar and co‐workers developed a different strategy for fluoride detection. They synthesized a triazole appended pentacenequinone derivative that can act together with Fe^3+^ as a fluoride turn‐on sensor.^12^ Since F^−^ has a stronger binding affinity to Fe^3+^ than the nitrogen atoms of triazole moieties, it can reactivate the fluorescence of the dye quenched by Fe^3+^ for detecting F^−^ with a limit of 3 μmol L^−1^, but the interference of AcO^−^ remains substantial. Herein, we show a novel fluorescent graphene oxide (GO) based fluoride sensor, where the nitrogen‐doped GO served not only as a substrate but also as a fluorogenic probe after its fluorescence completely quenched by Fe^3+^. A rapid and facile detection was subsequently enabled by turning on the quenched fluorescence with F^‐^, which even showed remarkable potentials for ultrasensitive visual detection through test paper.

Previous studies revealed that depending on the carbonization degree the pyrolysis of citric acid can lead to fluorescent graphene quantum dots and graphene oxide,^13^ while the involvement of ethylenediamine in the pyrolysis of citric acid greatly enhances fluorescence quantum yield of carbon dots up to ≈84%. 14 On the basis of these previous studies, herein highly fluorescent GO was prepared through reactions between citric acid and ethylenediamine in aqueous solution at elevated temperature, but in presence of ortho‐H_3_PO_4_ to facilitate the carbonization. Transmission electron microscopy (TEM) revealed that nanosheet is the dominant structure of purified product, as shown in **Figure** **1**a. The Raman spectrum of the nanosheets in Figure 1b presents two signals peaking at 1356 and 1606 cm^−1^, respectively, suggesting that the resulting nanosheets are GO as these two peaks are the typical D‐band and G‐band signals of GO. According to literatures, D‐band signal is caused by defects and broken symmetry of basal plane of graphitized carbon atom, and the G‐band is mainly due to the vibrational mode of sp^2^ hybridized carbon atoms in both rings and chain structures,^15^ while their shifts to higher wavenumber direction imply that the as‐prepared GO has nitrogen‐doping structure.^15^, ^16^ The integrated area ratio of the D‐band to G‐band signals is as high as 1.66, indicating that a numerous defects involving N atoms occur in the resulting structure since *I*_D_/*I*_G_ is inversely proportional to the size of sp^2^ carbon domains. Normally, the D‐band of undoped monolayers with a high degree of crystallinity is rather weak.^15^

**Figure 1:**
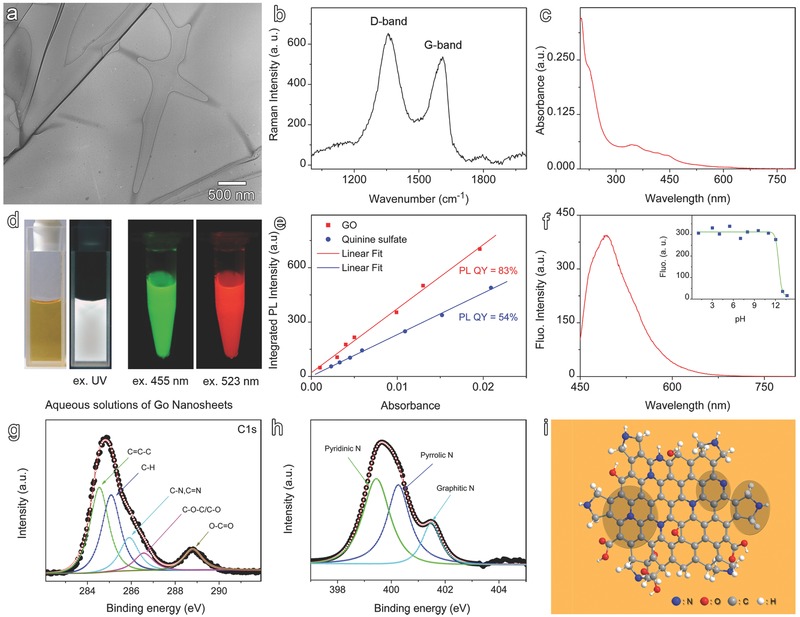
a) TEM image, b) Raman spectrum, and c) UV–vis absorption spectrum of as‐prepared N‐doped GO nanosheets. d) Optical images of aqueous solutions of GO nanosheets captured under room light (left), UV light (365 nm), and monochromatic lights (455 nm and 523 nm), respectively, for showing the excitation‐dependent emission. e) Fluorescence quantum yield measurements performed by comparing the integrated emissions of GO in water with those of Quinine sulfate in 0.1 m H_2_SO_4_ (the quantum yield of the latter was taken as 54% according to reference 14). f) Fluorescence spectrum of the as‐prepared GO nanosheets recorded with excitation at 420 nm, together with the intensities recorded at different pH values shown in the inset. g,h) C1s and N1s XPS spectra of GO nanosheets overlaid with the corresponding fits. i) Schematic illustration of GO nanosheets with structures involving pyridinic N, pyrrolic N, and graphitic N atoms being highlighted.

The absorption spectrum of the resulting GO nanosheets is presented in Figure 1c, in which the absorption bands at around 221 nm, 344 nm can be attributed to π–π* and n–π* transitions of GO, while the absorption band 442 nm can be attributed to nitrogen‐doping structures which were found to shift the absorption band of graphene quantum dots to the red.^17^

The brown GO nanosheets dispersed in aqueous solution present strong whitish fluorescence under UV excitation and remarkable excitation‐dependent emissions (Figure 1d) with a fluorescence quantum yield up to 83%, determined by using quinine sulphate as reference (Figure 1e). More quantitatively, the GO nanosheets exhibit abroad emission peaking at 492 nm upon excitation at 420 nm (Figure 1f) and this emission is relatively pH‐independent over a very large pH range from 1 to 12, as shown in the inset of Figure 1f and Figure S1 in the Supporting Information, which is very desirable for senor applications.

To provide more detailed structural information, careful X‐ray photoelectron spectroscopy (XPS) studies were carried out. The C1s signal shown in Figure 1g was fitted by five components with identical FWHMs (full width at half‐maximum), i.e., 1.00 eV, as given in Table S1 of the Supporting Information. Apart from the carbon atoms involving C—C=C bond in aromatic rings (284.6 eV), C—H bond in basal plane (285.0 eV), C—O bond in epoxy/hydroxyls groups (286.6 eV), and O—C=O bond in carboxyl groups (288.8 eV), which are typical graphene oxide, the signal peaking at 285.9 eV can be assigned to sp^2^ carbon in C=N bond according to reference.^18^ The as‐prepared sample also presents N1s signal that could be fitted with three signals locating at 399.4, 400.2, and 401.5 eV, respectively (Figure 1h and Table S2, Supporting Information). These three subpeaks can be attributed to pyridinic N, pyrrolic N, and graphitic N atoms,[[qv: 18b,19]] respectively, as illustrated in Figure 1i, but showing slight positive shifts in comparison with those of GO prepared through high temperature (≈800 °C) approaches,[[qv: 19b,c]] which can be understood by the formation of hydrogen bonding of N moieties as the current samples were directly synthesized in aqueous solution.^20^

It was found out that the fluorescence of the N‐doped GO can effectively be quenched by Fe^3+^ ions (**Figure** **2**a). As shown in the inset, the fluorescence intensity presents a linear relationship over two orders of magnitude against the concentration of Fe^3+^ ions on a log‐log plot, and nearly vanishes when the concentration of Fe^3+^ goes high enough. However, the fluorescence was found to be recoverable upon the introduction of F^−^ ion (Figure 2b). A good linear relationship between the relative emission intensity (*I*/*I*_0_) and F^−^ concentration, shown in the inset of Figure 2b, suggests that the N‐doped GO nanosheets can potentially be used for F^−^ sensing mediated by Fe^3+^.

**Figure 2:**
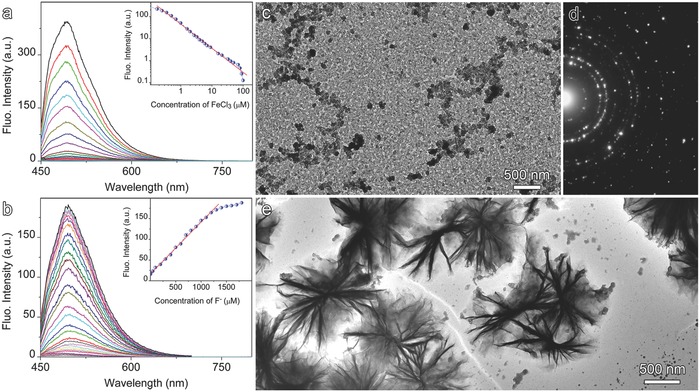
a) A set of fluorescence spectra of GO nanosheets recorded during titration with Fe^3+^ (inset shows fluorescence intensity against the concentration of FeCl_3_). b) A set of fluorescence spectra recorded during addition of F^−^ into aqueous solution of GO nanosheets with fluorescence initially quenched by Fe^3+^ (insets shows fluorescence intensity against the concentration of F^−^). c) TEM image of GO‐Fe (III) complexes d) together with selected area electron diffraction patterns which can be attributed to orthorhombic iron oxide hydrate according to JCPDS card (No. 3–0079) with more details being shown in Table S3 of the Supporting Information. e) TEM image of GO‐Fe (III) complexes captured after the fluorescence was recovered with F^−^.

To understand this fluorescence quenching/recovery phenomena, the morphology of GO nanosheets with its fluorescence being quenched by Fe^3+^ was characterized by TEM. A representative TEM image in Figure 2c reveals that Fe^3+^ ions form densely packed clusters on the surface of GO nanosheets, as reflected by the electron diffraction pattern in Figure 2d and Table S3 of the Supporting Information. This is because Fe^3+^ ions are very readily hydrolyzed to form nanocrystals of different forms. While the firm attachment of iron particles may be attributed to the interactions between electron‐donating N moieties of GO nanosheets with Fe (III) species^21^ and explains the fluorescence quenching effect of Fe^3+^.^21^, ^22^ However, after introducing F^−^ ions most clusters are liberated leaving wrinkled nanosheets behind (Figure 2e). This is because F^−^ ion is very readily to form stable complexes (FeF_x_)^n‐^ with Fe^3+^.^12^ Thus, it can be deduced that the adsorbed Fe^3+^ ions including those in nanoparticle forms are released upon introduction of F^−^ to reactivate the fluorescence of GO.

To verify the above hypothesis, the interactions between Fe (III) with GO were further investigated by XPS. The fitting results shown in **Figure** **3**a and Table S1 of the Supporting Information reveal that the C1s signal associated with C—N/C=N bonds shifts from 285.9 eV for the mother GO nanosheets (Figure 1g) down to 285.6 eV after forming complexes with Fe (III), while the following addition of F^−^ shifted this signal back up to 286.2 eV (Figure 3b). In the meantime, the signals associated with C—H, C—C=C, C—O—C, O—C=C remained nearly unchanged. All these facts strongly support that the fluorescence quenching takes place via the interaction of C—N/C=N moieties with Fe (III), which is better reflected by the N1s XPS results given in Figure 3c and Table S2 of the Supporting Information. In brief, the N1s signals at 399.4 eV (pyridinic N), 400.2 eV (pyrrolic N), and 401.5 eV (graphitic N) are positively shifted by 0.2, 0.1, and 0.4 eV upon introduction of Fe^3+^ owing to the decreased electron density of N atoms upon interaction with Fe^3+^ ions.[[qv: 19a]] However, upon the following introduction of F^−^ the appearance of two new signals at around 398.5 and 400.8 eV (Figure 3d) indicates the formation of partly recovered pyridinic N and graphitic N moieties.[[qv: 19a,b]] Even though not all N moieties are recovered, which explains the wrinkled structures leaving behind (Figure 2e) and incompletely recovered fluorescence after F^−^ addition (Figure 2a,b), the partly released pyridinic N and graphitic N moieties explains the recovery of the fluorescence of GO.

**Figure 3:**
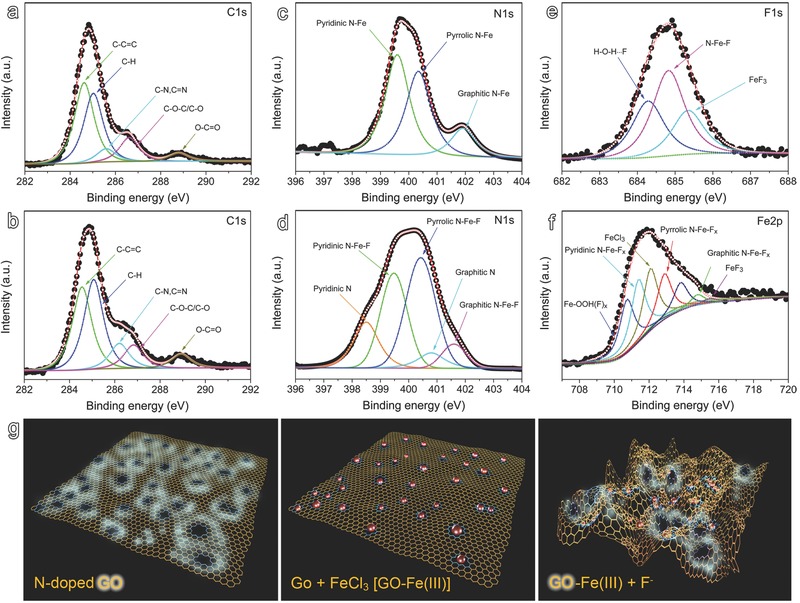
C1s, N1s, F1s, and Fe2p XPS spectra of GO‐Fe (III) complexes together with the corresponding fits recorded a,c) before and b,d,e,f) after the fluorescence was recovered by F^−^. g) Schematic drawing of N‐doped fluorescent GO nanosheets (left), GO‐Fe (III) complexes (middle), and nanosheets with fluorescence partly recovered with F‐ (right).

The fluorescence quenching/recovery mechanism can also find support from F1s XPS analysis (Figure 3e and Table S4, Supporting Information). After the fluorescence got recovered, the dried sample presented three F1s signals at 684.3, 684.8, and 685.3 eV, respectively. The signal at around 684.3 eV can be attributed to the formation hydrogen bonding in the form of H—O—H···F,^23^ while that at around 685.3 eV can be attributed to the formation of FeF_3_.^24^ The appearance of FeF_3_ strongly supports the mechanism mentioning above, as illustrated in Figure 3g. In addition, the signal at 684.8 eV is slightly shifted by +0.3 eV if compared with that for NaF (684.5 eV),^25^ which may be attributed to the formation of N—Fe—F bonding owing to the incomplete extraction of the adsorbed Fe (III) species remaining attached on GO nanosheets.^25^ In addition, Fe2p XPS analysis in Figure 3f and Table S5 of the Supporting Information also support the formation of FeF_3_ after the fluorescence recovery even though certain percentage of Fe (III) remaining attached to N moieties.^24^

To explore the potential of the fluorescence recovery of GO‐Fe (III) complexes for F^−^ sensing, various types of anions such as Cl^−^, Br^−^, I^−^, SO_4_^2−^, NO_2_^−^, NO_3_^−^, HCO_3_^−^, HCOO^−^, CO_3_^2−^, PO_4_^3−^, and CH_3_COO^−^ in the form of sodium salts were adopted for examining the selectivity of F^−^ detection. The fluorescence measurements in **Figure** **4**a and Figure S2 of the Supporting Information reveal that only sodium fluoride can effectively recover the fluorescence of GO‐Fe (III) complexes (0.1 mg mL^−1^), whereas the rest anions only lead to much weaker fluorescence recovery, verifying the outstanding selectivity of GO‐Fe (III) complexes for F^−^ detection. Moreover, as shown in Figure S3 of the Supporting Information the collective contribution of the above 11 types of anions to the recovered fluorescence is only 5.5% of that achieved by F^−^ of 0.1 × 10^−3^ m which is slightly higher than enforceable drinking water standard for fluoride (i.e., 1.2 mg L^−1^) given by United States Environmental Protection Agency. All these results suggest that GO nanosheets together with Fe^3+^ form an excellent system for sensitive F^−^ detection. In fact, most F^−^ detection methods face selectivity problems especially for excluding the interference of anions such as HCOO^−^, PO_4_^3−^, and CO_3_^2‐^ due to their similar basicity,^26^ but the current system well overcomes these limitations.

**Figure 4:**
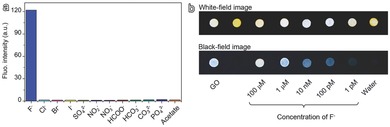
a) Fluorescence intensity of GO‐Fe (III) complexes in a series of aqueous solutions containing equal molar anions of different types for testing their ability to recover the quenched fluorescence. b) Photographs of a series of spherical filter papers stained with GO‐Fe (III) complexes, captured under room light (top), and UV light (bottom), respectively, after differently concentrated F^−^ solutions were dripped on each test paper for illustrating the visual detection limit of F^−^ ions.

To further show the potential application of sensitive F^−^ detection, filter papers stained with the GO‐Fe (III) complexes were prepared, dried, and then the fluorescence of each stained spots were imaged under UV irradiation (365 nm) following the introduction of F^−^ in different concentrations, for proof‐of‐concept tests of visual F^−^ ion detection. As presented in Figure 4b, no observable fluorescence can be observed from the spot of GO‐Fe (III), but the fluorescence can easily be identified even when the concentration of F^−^ ion goes down to 1 × 10^−12^ m. Although the color of the stained spots is slightly lightened by different degrees, owing to the drifting of excess Fe (III) complexes by the F^−^‐containing water droplets to surrounding, the F^−^ concentration‐dependent fluorescence recovery tendency is not affected if compared with that induced by pure water. It is also deserved to mention that the exposure time for taking the white‐field and black‐field images was only of 1/60 and 1/2 s, respectively, with an amateur digital single lens reflex camera. More importantly, the fluorescence intensity recovered by differently concentrated F^−^ can easily be differentiated by naked eyes. In addition, the fluorescence recovery completed so quickly that no following fluorescence variation was practically observed a few tens of seconds after the addition of F^−^ droplets. Thus the current system offers an efficient and facile approach for monitoring the F^−^ content of water systems. Even though, the testing paper is just a proof of concept design for convenient and ultrasensitive detection of F^−^ ion in aqueous media, the above results are very encouraging for developing on‐site detections required in fields of environment, medicine, food, etc.

In summary, a simple sensing method for selective recognition and rapid detection of fluoride anions has been established by using highly fluorescent GO nanosheets. The key finding together with in‐depth XPS studies clearly reveal that via the interactions between the N moieties of N‐doped GO nanosheets and Fe^3+^ a fluorescence‐quenching system can be established, while through the interaction between F^−^ and Fe (III) quenchers the fluorescence of GO can effectively be reactivated. Since F^−^ exhibits the highest binding constant for forming stable complexes with Fe (III) among most anions commonly present in aqueous media, the fluorescence can solely be recovered by F^−^ ion, thus enabling effective and ultrasensitive detections of F^−^ ions, which is demonstrated by proof of concept experiments through testing paper‐based visual detection with limit down to 1 × 10^−12^ m. In brief, the extremely low visual detection limit, excellent anti‐interference of commonly occurring anions, together with the inexpensive materials make the GO‐Fe (III) system potentially valuable for onsite F^−^ detection for industry.

## Supporting information

As a service to our authors and readers, this journal provides supporting information supplied by the authors. Such materials are peer reviewed and may be re‐organized for online delivery, but are not copy‐edited or typeset. Technical support issues arising from supporting information (other than missing files) should be addressed to the authors.

Supplementary
