# Supplementary material for: Graphene Oxide‐Based Sensor for Ultrasensitive Visual Detection of Fluoride
Source: Adv Sci (Weinh). 2016 Jul 21;3(12):1600217. doi: 10.1002/advs.201600217 (PMC5157177; doi:10.1002/advs.201600217)
Supplement: Supplementary file 1 — Supplementary [file ADVS-3-0-s001.pdf]

Copyright WILEY-VCH Verlag GmbH & Co. KGaA, 69469 Weinheim, Germany, 2016.

ADVANCED SCIENCE
Open Access

Supporting Information

for *Adv. Sci.,* DOI: 10.1002/advs.201600217

Graphene Oxide-Based Sensor for Ultrasensitive Visual Detection of Fluoride

*Tapas K. Mandal, Yi Hou, Zhenyu Gao, Haoran Ning, Wensheng Yang, and Mingyuan Gao**

Supporting Information

Graphene Oxide-based Sensor for Ultrasensitive Visual Detection of Fluoride

Tapas K. Mandal[#,1], Yi Hou[#,1], Zhenyu Gao[1,2], Haoran Ning[1], and Mingyuan Gao[*,1]

[1]*Institute of Chemistry, Chinese Academy of Sciences, Bei Yi Jie 2, Zhong Guan Cun, Beijing 100190, China, University of Chinese Academy of Sciences, Beijing 100049, China,* [2]*College of Chemistry, Jilin University, Changchun 100032, China.*

Experimental Section

The N-doped GO nanosheets were prepared by modifying a literature method[16, 37]. In a typical synthesis, 2 g of citric acid was firstly dissolved in 3 mL water and then mixed with 10 mL of ortho-H_3PO_4 (85%). After that, the reaction mixture was heated to boiling under open air condition for 15 min, into which 1 mL NaOH (1 M) solution and 80 mg 1-ethyl-3-(3-dimethylaminopropyl) carbodiimide were subsequently added to activate the carboxyl groups. During the following 30 min reaction, the colour of the reaction mixture changed from colourless to pale yellow. Then, 400 µL of 1,2-ethylenediamine was introduced. Upon prolonged reaction, the colour of the solution gradually changed from yellow to brownish-black. The reaction was terminated after 5 h by naturally cooling the reaction system down to room temperature. Upon introduction of 50 mL ethanol, brownish-black precipitate was generated and collected by centrifugation at 7000 rpm. The brown product was purified by being washed for several times with ethanol followed by the centrifugation to achieve purified N-doped GO nanosheets which were highly dispersible and fluorescent in water.

The fluorescence quenching experiments were carried out as follows. Typically, 10 mg GO was dissolved in 100 mL Milli-Q water. Under continuous stirring, 0.1 mM $FeCl_3$ solution was added dropwise to quench the fluorescence. Until no fluorescence was detected, indicating the formation of GO-Fe (III) complexes, 0.1 mM NaF solution was slowly introduced into the solution to recover the fluorescence. During the whole process, the fluorescence of the solution was monitored with fluorescence spectrophotometer.

For the test paper experiments, the GO-Fe(III) solution was dripped on lab filter paper. After the stained spots got dried under ambient conditions, F^- solutions was carefully dripped to the centre of each spots. Approximately 2 min later, the paper strips with a series of stained spots were imaged with a SLR camera (Nikon D 7000) equipped with an AF-S Nikkor lens (18-200 mm 1:3.5-5.6 GII ED). The imaging parameters were set as follows: Aperture, f/7.1; exposure time, ½ S; ISO, 1600).

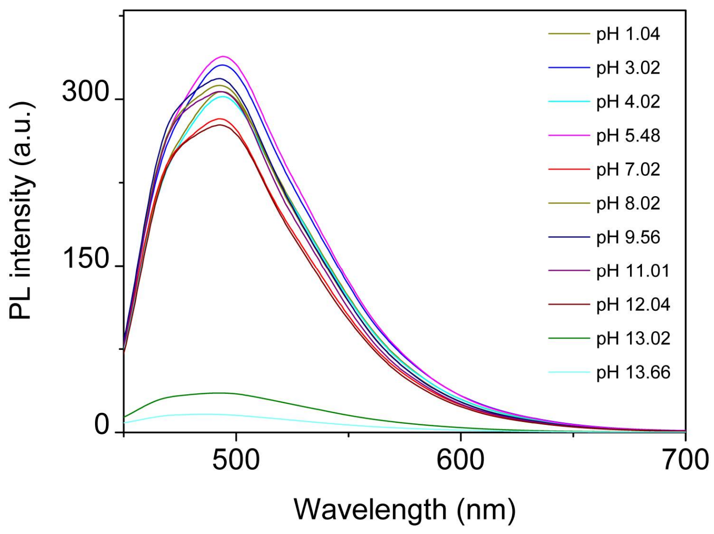

Figure S1. Fluorescence spectra of GO nanosheets at different pH.

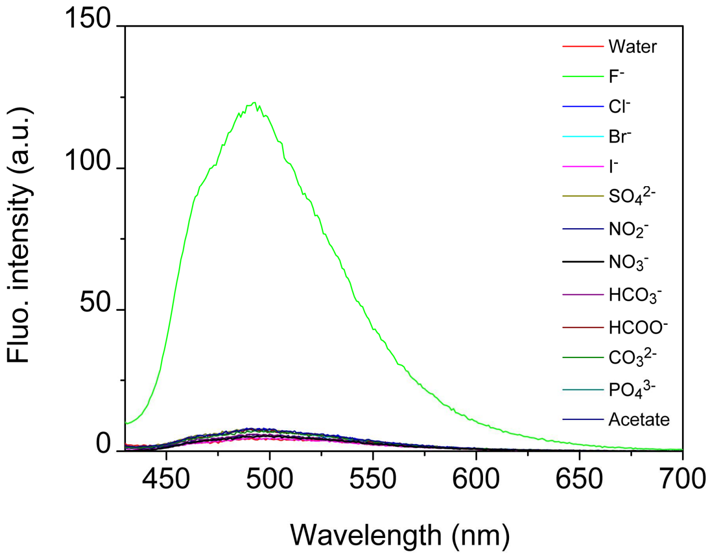

Figure S2. Fluorescence spectra of GO-Fe (III) complexes in pure water or aqueous solutions containing different types of anions (0.1 mM), respectively.

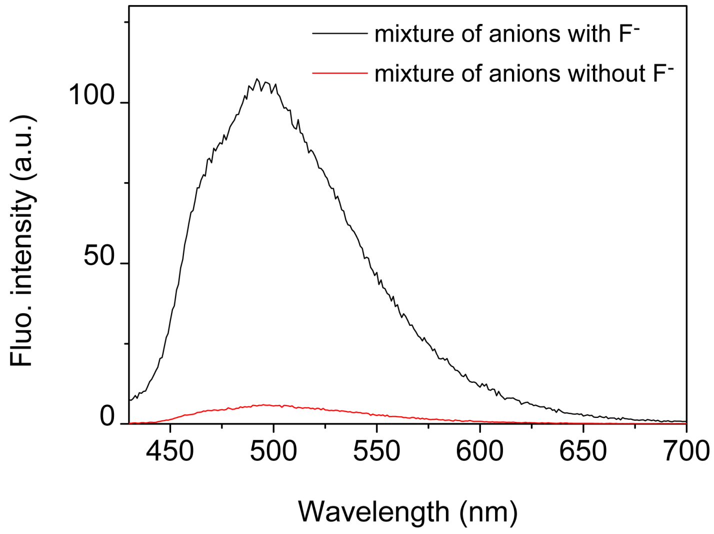

Figure S3. Fluorescence spectrum of GO-Fe (III) complexes in aqueous solution containing F^-, Cl^-, Br^-, I^-, SO_4^{2-}, NO_2^-, NO_3^-, HCO_3^-, $HCOO^-$, CO_3^{2-}, PO_4^{3-}, and CH_3COO^- for comparison with that recorded in the absence of F^-. The concentration of all anions invovled is 0.1 mM.

Table S1. Detailed fitting results of C1s XPS signals

GO		GO-Fe (III) complexes		GO-Fe (III) complexes + F^-		FWHM (eV)	Chem. states
B.E. (eV)	Area (%)	B.E. (eV)	Area (%)	B.E. (eV)	Area (%)		
284.6	36.21	284.6	41.29	284.6	35.29	1.00	C-C=C[1]
285.0	32.52	285.0	35.49	285.0	38.19	1.00	C-H[2]
285.9	14.52	285.6	6.74	286.2	10.67	1.00	C-N, C=N[1-3]
286.6	7.62	286.6	12.23	286.8	9.89	1.00	C-O-C/C-O[1-3]
288.8	9.13	288.8	4.25	288.9	5.96	1.00	O-C=O[1]

Table S2. Detailed fitting results for N1s XPS signals

B.E. (eV)	Area (%)	FWHM (eV)	Chemical state
GO			
399.4	45.64	1.000	Pyridinic N[3]
400.2	40.38	1.000	Pyrrolic N[3, 4]
401.5	13.98	1.000	Graphitic N[1, 5, 6]
GO-Fe (III) complexes			
399.6	46.58	1.000	Pyridinic N-Fe[3]
400.3	40.42	1.000	Pyrrolic N-Fe[3, 7]
401.9	13.00	1.000	Graphitic N-Fe[5, 7]
GO-Fe (III) complex + F^- ion			
398.5	15.44	1.000	Pyridinic N[1, 3]
399.5	30.32	1.000	Pyridinic N-Fe-F[1, 3]
400.3	40.56	1.000	Pyrrolic N-Fe-F[3, 4]
400.8	5.91	1.000	Graphitic N[8]
401.6	7.77	1.000	Graphitic N-Fe-F[1, 5, 6]

Table S3. Comparison of SAED data of GO-Fe (III) complexes with the standard JCPDS card data for orthorhombic iron oxide hydrate (No. 3-0079)

	(211)	(060)	(031)	(011)
**Calculated d value (Å)**	1.65	2.12	2.45	2.96
**Standard d value (Å)**	1.63	2.09	2.48	2.99
**deviation**	1.2%	1.2%	-1.4%	-1.1%

Table S4. Detailed fitting results for F1s XPS signals

GO-Fe (III) Complexes + F^- ion			
**B.E. (eV)**	**Area (%)**	**FWHM (eV)**	**Chemical state**
684.3	30.4	1.00	H-O-H···F[10]
684.8	45.9	1.00	N-Fe-F[10]
685.3	23.7	1.00	FeF_3[10]

Table S5. Detailed fitting results for Fe2p XPS signals

GO-Fe (III) Complexes + F^- ion			
**B.E. (eV)**	**Area (%)**	**FWHM (eV)**	**Chemical state**
710.7	8.1	1.00	Fe-OOH$(F)_x$[9]
711.5	17.3	1.00	Pyridinic N-Fe-F_x[7]
711.9	28.0	1.00	$FeCl_3$[9]
712.9	20.5	1.00	Pyrrolic N-Fe-F_x[7, 9]
713.9	16.5	1.00	N/I
714.9	6.6	1.00	Graphitic N-Fe-F_x[7, 9]
715.6	3.0	1.00	FeF_3[9]

References:

1. Mou, Z. *et al.* Forming mechanism of nitrogen doped graphene prepared by thermal solid-state reaction of graphite oxide and urea. *Appl. Surf. Sci.* **258**, 1704-1710 (2011).
2. Yang, D. *et al.* Chemical analysis of graphene oxide films after heat and chemical treatments by X-ray photoelectron and Micro-Raman spectroscopy *Carbon* **47**, 145-152 (2009).
3. Artyushkova, K. *et al.* Density functional theory calculations of XPS binding energy shift for nitrogen-containing graphene-like structures. *Chem. Commun.* **49**, 2539-2541 (2013).
4. Kelemen, S. R., Gorbaty, M. L. & Kwiatek, P. J. Quantification of nitrogen forms in Argonne premiumcoals. *Energy & Fuels* **8**, 896-906 (1994).
5. Wang, L. *et al.* Nitrogen, cobalt-codoped carbon electrocatalyst for oxygen reduction reaction using soy milk and cobalt salts as precursors. *Electrochem. Commun.* **34**, 68-72 (2013).
6. Titantah, J. T. & Lamoen. D. Carbon and nitrogen 1s energy levels in amorphous carbon nitride systems: XPS interpretation using first-principles. *Diam. Relat. Mater.* **16**, 581-588 (2007).

7. Ebner, J. R., Mcfadden, D. L., Tyler, D. R. & Walton, R. A. X-ray photoelectron spectra of inorganic molecules. 12. Chlorine 2p binding energies of dinuclearchloro anions and mononuclear chlorocations of the transition metals. *Inorg. Chem.* **15**, 3014-3018 (1976).
8. Wang, J., Lin, X. Y., Luo, X. G. & Long, Y. F. A sorbent of carboxymethyl cellulose loaded with zirconium for the removal of fluoride from aqueous solution. *Chem. Eng. J.* 252, 415-422 (2014).
9. Lu, W. *et al.* A novel chemosensor based on Fe (III)-complexation for selective recognition and rapid detection of fluoride anions in aqueous media. *Tetrahedron* **67**, 7909-7912 (2011).
10. Grosvenor, A. P., Kobe, B. A., Biesinger, M. C. & McIntyre. N. S. Investigation of multiplet splitting of Fe 2p XPS spectra and bonding in iron compounds. *Surf. Interface Anal.* **36**, 1564-1574 (2004).
